# LPMX: a pure rootless composable container system

**DOI:** 10.1186/s12859-022-04649-3

**Published:** 2022-03-31

**Authors:** Xu Yang, Masahiro Kasahara

**Affiliations:** Department of Computational Biology and Medical Sciences, The University of Tokyo, Tokyo, Japan

**Keywords:** Virtualization, Container system, Pipelines

## Abstract

**Background:**

Delivering tools for genome analysis to users is often difficult given the complex dependencies and conflicts of such tools. Container virtualization systems (such as Singularity) isolate environments, thereby helping developers package tools. However, these systems lack *mutual composability*, i.e., an easy way to integrate multiple tools in different containers and/or on the host. Another issue is that one may be unable to use a single container system of the same version at all the sites being used, thus discouraging the use of container systems.

**Results:**

We developed LPMX, an open-source pure rootless composable container system that provides composability; i.e., the system allows users to easily integrate tools from different containers or even from the host. LPMX accelerates science by letting researchers compose existing containers and containerize tools/pipelines that are difficult to package/containerize using Conda or Singularity, thereby saving researchers’ precious time. The technique used in LPMX allows LPMX to run purely in userspace without root privileges even during installation, thus ensuring that we can use LPMX at any Linux clusters with major distributions. The lowest overhead for launching containers with LPMX gives us courage to isolate tools as much as possible into small containers, thereby minimizing the chance of conflicts. The support for the layered file system keeps the total size of container images for a single genomic pipeline modest, as opposed to Singularity, which uses mostly a flat single-layer image.

**Conclusions:**

LPMX is pure rootless container engine with mutual composability, thus saving researchers’ time, and accelerating science.

## Background

Genome analysis usually involves shepherding data files by using many tools and scripts, called pipelines or workflows [1–4]. As genome analysis becomes more complex, more third-party packages and tools are needed, and conflicting packages, including different versions of the same package, are more likely to exist. This situation is known as dependency hell. Tools used in genome analysis require variety of environments, such as dependent libraries or compiler versions. Setting up environments often takes researchers a long time and thus discourages researchers from using hard-to-install programs regardless of the programs’ scientific merits, thereby making the progress of genome science significantly slower than necessary. Dependency hell is an urgent problem to address in genomics.

Community efforts, such as Bioconda [5], virtually eliminated the software installation problem for users; eliminating this problem is an enormous achievement. However, creating a Conda package sometimes requires more time than creating a container image does, especially when distro-specific configurations (e.g., the existence of pkg-config, apt) are required to build a third-party package.

This issue is easily mitigated when the filesystem namespace is isolated, as with container-based virtualization systems, such as Singularity [6], Docker [7] or udocker [8]. These systems enable developers to use their favorite Linux distributions without struggling to make a program (and its build procedure) compatible with the Conda build environment. We define *modularity* of a package by having a separate filesystem namespace for that package. Container-based virtualization provides modularity, which helps developers more easily package and distribute new programs.

However, we lose a certain level of productivity when we migrate from the Conda environment to containers. This loss is due to the loss of *composability*, which we discuss shortly. In other words, researchers often have to spend more time in algorithm development when they use containers rather than the Conda environment.

With Conda, we can write programs that call (by exec$*$ functions) programs either in Conda packages or on the host, and we can also package these programs as new Conda packages because programs on the host and in Conda packages can call each other; we call this ability *mutual composability*. In contrast, it is difficult for programs in containers to call (by exec$*$ functions) a program on the host or a program in another container due to the namespace isolation; we need either to make programs aware of containers or to write wrapper scripts for the programs to keep using exec$*$ functions.

For example, we can write experimental one-off Bash-script-based analysis pipelines that call binaries in Conda packages. When we migrate to container-based solutions, the analysis pipelines must be significantly re-engineered such that they call a container engine instead of directly calling binaries. An environment manager, such as Bulker [9], helps us generate wrapper scripts so that we can call programs in containers as if they are programs on the host. However, once we take this approach, we cannot easily containerize the analysis pipelines themselves because programs in containers cannot directly call (by exec$*$ functions) programs in other containers. Thus, Bulker achieves only (what we call) *one-way composability*, not mutual composability. The lack of mutual composability may become an issue in other situations. For example, the Canu assembler [10] is difficult to package by using current container systems because Canu inside a container cannot submit jobs (for parallelization) to the batch job engine, which is outside the container, while Conda does not suffer from this issue.

**Fig. 1:**
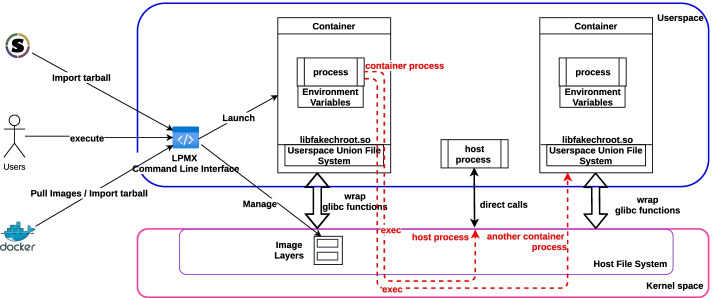
An overview of the LPMX system architecture. LPMX contains a command line interface (CLI) and a dynamic library wrapping filesystem-related standard C library functions and faking the file system to provide its features. LPMX can manage the life cycles of created containers and download, import and maintain external images and tarballs. By configuring containers mappings, such as executable mapping, processes that reside inside containers can make direct calls to processes on the host or in other containers

Other issues with popular container systems in high-performance computing (HPC), such as Singularity, are (1) users cannot create a new container image on HPC because they do not have root privileges, (2) users hesitate to containerize small genomics tools because a Singularity image must contain a full set of operating system (OS) images even if the target tool is very small, and (3) when users have accounts on multiple HPC sites, some sites completely lack container systems, or other sites may have Singularity but with different major versions, thus making the users create many Singularity images, even for a single tool. Pure rootless container engines, such as udocker, solve problem (1) and (3), while container engines that support layered file systems (e.g., Docker) solve problem (2). However, none of the existing systems solve all of the problems simultaneously.

Here, we propose a new rootless container engine, LPMX, that solves all these issues. LPMX provides mutual composability for letting a container interface easily with the host or with another container while maintaining modularity. LPMX runs without root privileges during runtime or during installation, thus providing users with full features at any Linux cluster without administrators’ approval. LPMX has the first layered file system that, unlike an existing similar file system, FUSE-overlayfs [11], is implemented purely in userspace. Figure 1 shows an overview of the LPMX system architecture, where LPMX manages the life cycles of created containers and external images. LPMX can provide mutual composability for letting processes inside containers make direct calls to processes on the host or in other containers.

## Implementation

The primary goals of LPMX are a) to provide composability over containers and hosts, b) to provide pure rootless containers, and c) to support a layered file system.

To create a pure userspace container system, we developed a fake chroot environment based on fakechroot [12], a project giving a chroot-like environment to end-users (nonroot) by employing the LD_PRELOAD hack. The LD_PRELOAD hack enables us to inject arbitrary functions into dynamic libraries. Because we inject special wrapper functions, tools see a fake virtual file system that does not truly exist. For example, when a tool tries to open a file, an injected function replaces the path (in a container) provided by the tool with a path to the real file on the host, thus enabling processes in the container to open files in the container without using root privileges. Using this technique, we developed a pure userspace layered file system, namely, the Userspace Union File system (UUFS), and integrated it into LPMX. The underlying data structure of UUFS is compatible with Docker, so we can easily import Docker images while retaining layers.

The composability is implemented in LPMX by wrapping exec* functions in the GNU C library with the LD_PRELOAD hack. When a process inside the container calls an exec* function, LPMX traps the function, and if the callee is one of the list of executables to compose, LPMX redirects the exec call to the target executable, which might be on the host or in another container.

Like Docker, Singularity, and udocker, LPMX also has a specific general-purpose graphics processing unit (GPGPU) support. Feature comparison Table 1 lists the key features of LPMX compared to existing popular implementations.

**Table 1:** Feature comparison between major container systems and virtual machines (VM)

Features	LPMX	Docker	Singularity	VM	udocker	podman
Composability^a^	Y	N	N	N	N	N
Pure rootless^b^	Y	N^e^	N	N	Y	N^e^
Use docker image	Y	Y	Y	N	Y	Y
Use singularity image	Y	N	Y	N	N	N
Support layered filesystem	Y	Y	N	N	N	Y
Run programs statically linked to glibc	N^d^	Y	Y	Y	Y	Y
GPGPU support	Y	Y	Y	Y	Y	Y

^a^Allow processes in one container to make exec calls to other processes on the host or in other containers via standard posix application programming interface (API), such as posix_spawn

^b^Do not require root/sudo privileges in any stage, such as when installing a container system and its dependencies, nor when creating containers

^c^Singularity supports only up to two layers, but creating and using an overlay layer requires root privileges in practice

^d^Proprietary software is occasionally statically linked to glibc

^e^Podman and rootless Docker themselves can be installed without root privileges, although installing and configuring dependencies, such as uidmap, and some kernel parameters require root privileges on major Linux distributions in HPC

## Results

### Example of composing a container with executables on the host

To demonstrate the composability of LPMX, we containerized Canu [10] assembler (ver 2.1.1), a popular *de novo* assembler for long reads. The Canu assembler can automatically detect major batch job systems, e.g., Univa Grid Engine (UGE), and configure itself to work with the batch job system for distributed execution. If we naïvely containerize the Canu assembler, the containerized Canu assembler would try to find a batch job engine inside the container; however, the batch job engine is on the host, which is outside the container. We would have to create a host daemon that receives requests from inside the container via TCP sockets so that the Canu assembler can submit new jobs to the batch job engine. Creating such a daemon is, in theory, possible, but requires significant engineering. Another problem with this approach is that we need to modify the container image, which is often provided by third parties; once we modify anything in the container image, we need to keep up with upstream changes forever, and this need is a large burden. We need a way to keep up with upstream changes without modifying any files in the container to avoid future maintenance burden. LPMX provides a way to allow processes inside a container to call executables on the host or executables on another container; we call this ability *composability*.

**Fig. 2:**
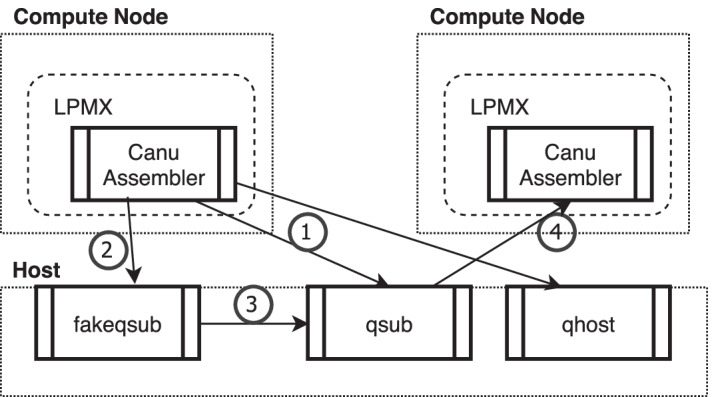
Example of composing a host process and container. (1) The Canu assembler uses batch commands (e.g., qhost) to query information from the job engine system. The Canu assembler container image is not modified, but LPMX transparently redirects the commands to the host batch job engine. (2) When submitting a job (e.g., use qsub), the command is trapped by LPMX and redirected to fakeqsub, which is a delegate program that generates a shell script to restore containers on other compute nodes. (3) Then, fakeqsub calls the host qsub to submit the generated shell script. (4) When the generated shell script is executed on other compute nodes, the LPMX container is resumed, and the containerized Canu assembler is launched. These redirections occur based on the LPMX configuration so that the container image and the Canu assembler do not require modification, which makes it easier to keep up with upstream changes

Due to the lack of composability, traditional container systems, such as Singularity, cannot be used to containerize the Canu assembler without modifying files inside images. LPMX readily solves this issue (Fig. 2). This example shows that the containerized Canu assembler can readily directly call executables on the host, and that the Canu assembler remains fully workable (distributing computation to many nodes in parallel) inside LPMX thanks to composability.

To show that the composability feature of LPMX works as expected, we used *E. coli* K12 Oxford Nanopore reads^1^ with the Canu assembler on the supercomputer SHIROKANE,^2^ equipped with UGE, in the Human Genome Center (HGC). LPMX successfully distributed computation to multiple nodes. The version of LPMX we used in this manuscript is alpha-1.6.3.

### Example of composing a container with other containers

In exploratory experiments in genomics, combinations of different tools and different versions of the same tools are tested to obtain better results. With previous container systems, researchers have to spend considerable time and effort containerizing all combinations manually into a single container. If every tool in the pipeline is isolated as a single container, again, we need to develop a glue daemon that connects one container with another. Additionally, we need to modify programs inside containers so the programs transmit data and requests to another program in another container due to the lack of composability. Once we go in this direction, we suffer from the maintenance burden. We do not wish to modify any files inside container images provided by third parties. LPMX can map executables in one container to certain paths in another container so executables in the former can call executables in the latter. When an executable in the latter is called, the executable is launched in a new container with the container image of the latter; in this way, we can isolate tools as much as possible while letting users develop a pipeline that uses potentially conflicting tools without concerns.

**Fig. 3:**
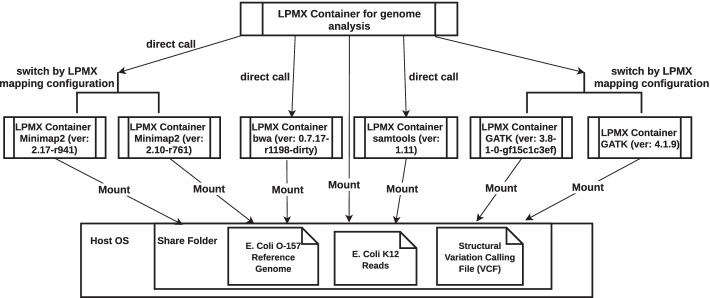
An overview of the container-container composability experiment. We first separated and containerized different tools into different containers to mimic the situation where users do not want to edit anything inside containers to exploit existing container images. Next, we created a pipeline container (without any analysis tools installed) by using the composability of LPMX. The pipeline container is composed from existing containers (tools) as if they were installed locally in the pipeline container. Consequently, the user can download a third-party pipeline image and plug different versions of the tools used in the original image to see the difference without modifying files inside the original image; to our knowledge, LPMX is the only tool that provides us with container-container composability. All containers shared the same directory in the host to allow data access and exchange. We put the *E. coli* K12 and *E. coli* O-157 datasets into the shared folder so that all containers could access it

To demonstrate the composability over multiple containers, a structural variation calling experiment on the reads of *E. coli* O-157 (SRR5383868) against the reference genome dataset *E. coli* K12 MG1655 (GCA_000005845.2) consisting of using different tools was performed. This experiment was conducted using a virtual machine created by VirtualBox (ver 6.0.24, employing Vagrant), and the virtual machine file and experiment scripts are available on Vagrant Cloud^3^ for reproducibility. We composed different containerized tools, including bwa (ver 0.7.17-r1198-dirty) [13], minimap2 (ver 2.10-r761 & 2.17-r941) [14], samtools (ver 1.11) [15], and different versions of the Genome Analysis Toolkit (GATK) (ver 3.8-1-0-gf15c1c3ef & 4.1.9) [16], into a pipeline container for testing various combinations of the software. Figure 3 shows that LPMX allowed end users to combine arbitrary containers; users can easily replace a tool used in a pipeline container with a newer (or older) version to see how the result would change without changing any files/directories inside existing containers.

### Container creation and destruction speed benchmark

**Fig. 4:**
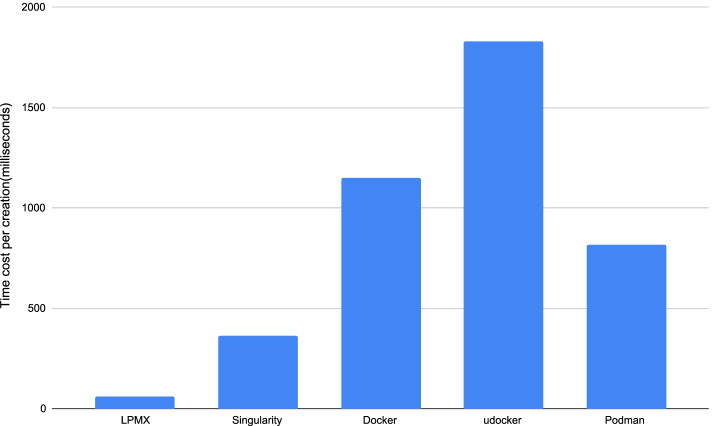
Average time (in ms; lower is better) required to create a container by several container systems. The compared container systems are: Docker (version: 19.03.6), podman (version: 1.6.2), Singularity (version: 3.7.0), udocker (version: 1.1.4), LPMX (version: alpha-1.6.3). The host was equipped with an Intel i7-8750H processors @2.2GHz (6 cores and 12 threads) and 32 GB RAM memory. The virtual machine was version 6.1. The virtualized host was Ubuntu 18.04.5 LTS (Bionic Beaver). The memory size was 1024 MB. The containerized Guest OS was also Ubuntu 18.04 LTS (Bionic Beaver) downloaded from DockerHub by using the tag Ubuntu:18.04. A shell script was executed in the virtualized host OS to evaluate the performance. The script creates and then immediately destroys the container and measures time cost. The *time* command measures the resource usage of a given running command and outputs the elapsed real time between invocation and termination, the user CPU time, and the system CPU time. We summed the user and the system CPU times to obtain the total time required to run the userspace logic code and execute the kernel space system calls during the run

UUFS in LPMX has a small overhead for launching a new process. To compare the container creation and destruction speed of LPMX (ver alpha-1.6.3) to those of Docker (ver 19.03.6), Singularity (ver 3.7.0), udocker (ver 1.1.4) [8], and podman (ver 1.6.2) [17], we created and destroyed a container ten times and measured the associated time cost by using the shell built-in *time* command. The time for launching a new process is reduced by up to 6-fold for LPMX compared to other implementations (Fig. 4). We used a virtual machine using VirtualBox (employing Vagrant) with experimental materials.^4^$$^,$$^5^ The experiment was repeated five times, and the data were averaged to represent a more accurate result. LPMX can minimize the overhead of splitting a large pipeline into smaller containerized components or tools to avoid conflicts between the components. A caveat is that compared to Singularity, the LPMX approach might put a larger burden on a central shared file system, so Singularity might scale well beyond a certain large number of nodes.

### Acceptable runtime overhead on a supercomputer

The LD_PRELOAD hack substantially wraps the original default (glibc) functions and executes extra code in the userspace, thereby possibly triggering additional system calls and causing some performance overhead. However, the read/write functions are kept untouched inside LPMX; these functions are frequently called in genome analysis and will not be slowed down. We performed a structural variant calling analysis on the HG002 human genome reads from NIST’s Genome in a Bottle (GIAB) project (sequencing depth: 30x) against GRCh38 Genome Reference Consortium Human Reference 38 and measured the time on the supercomputer SHIROKANE in the Human Genome Center located at the Institute of Medical Science, the University of Tokyo. We could not observe performance overhead larger than variance over multiple experiments. We ran the experiments five times and calculated the averages. The results suggest that the performance overhead in real-world genome analysis pipelines is acceptable.

**Table 2:** Running time for bwa-mem2 (ver 2.2.1), samtools (ver 1.13 with htslib) and GATK (ver 4.2.1.0 with HTSJDK ver 2.24.1 Picard ver 2.25.4) with the same dataset on the bare host and LPMX

Tool	Bare Host (min)		LPMX (min)	
bwa-mem2	162.1	± 9.7	150.4	± 4.5
samtools	495.9	± 93.8	446.1	± 6.0
GATK4	854.5	± 68.5	864.6	± 70.2

For GATK, we record the time for HaplotypeCaller. During the experiment, the shared file server is moderately loaded. The time difference between the bare host and LPMX can be explained largely by varying loads of the central shared file system over time, not by the performance overhead of LPMX. Data files and read files are available here (https://github.com/genome-in-a-bottle/giab_data_indexes/blob/master/AshkenazimTrio/sequence.index.AJtrio_Illumina300X_wgs_07292015.HG002, https://hgdownload.soe.ucsc.edu/goldenPath/hg38/bigZips/). The scripts are available here (https://github.com/JasonYangShadow/experiment_attachments)

### Benchmark on worst-case runtime overhead of LPMX

**Fig. 5:**
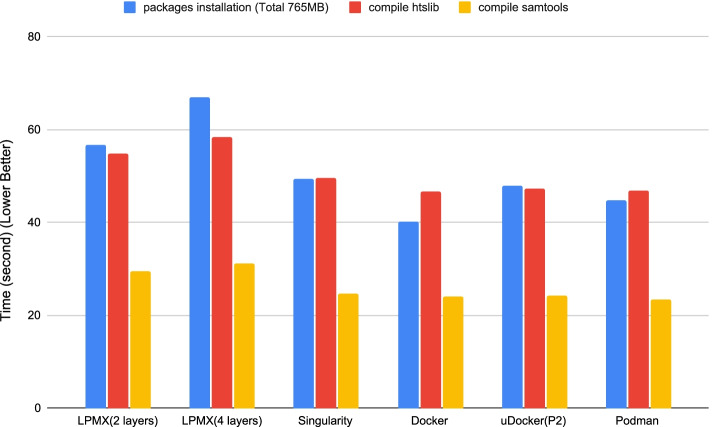
Average time when installing and compiling the same packages in different container systems. Typically, LPMX exhibits performance overhead slightly more than other implementations. Reducing the search layer depth reduces the overhead

A possible criticism against the UUFS architecture that adopts pure userspace implementation might be that metadata operations are slow. Here, we designed a performance benchmark test to reveal the worst performance overhead case of LPMX compared to other implementations. The experiment was designed and executed inside the same environment used in the section. As software installation imposes a heavy I/O in genome analysis, we measured the overhead thereof. The root reason for performance overhead is caused by the execution of userspace logic code and extra system calls in kernel space. To evaluate the performance overhead of LPMX, common software and packages (such as GCC, Python3, Ruby and Java Runtime Environment (JRE)), are installed, and the most popular genome analysis tools and their dependencies, (i.e., samtools and htslib) are compiled. The versions of all software are the same as those in the previous sections. The experiments were repeated five times and the average time costs measured by the built-in *time* command were calculated. As shown in Fig. 5, with respect to installing and compiling packages, the performance overhead of LPMX was 1.5 times greater than that of the other container system implementations. However, users usually need to install software only once inside containers; therefore, the performance overhead suffers only once. However for actual analysis repeatedly executed inside containers, only minimal performance overhead was observed as shown in Table 2, because functions, e.g., read/write, frequently called in the analysis are unwrapped in LPMX so that the raw performances are preserved.

### GPGPU support

GPGPUs are becoming more popular as more genomics applications are optimized appropriately. Docker, Singularity, and udocker support GPGPU. We thus added GPGPU support to LPMX. As LPMX can expose arbitrary files and directories on the host to a container, we exposed GPGPU-related files (especially special devices) to containers. To test whether GPGPUs can be used inside containers, we ran Guppy, a proprietary basecaller for Oxford Nanopore sequencers using GPGPUs. Guppy successfully used GPGPUs inside an LPMX container and we observed that there were no difference in outputs and the processing speed, as we expected. We executed the experiment five times and calculated the average values. As shown in Table 3, we observed no performance overhead.

**Table 3:** Performance information returned by the Guppy basecaller (ver 3.4.5+fb1fbfb) at the end of the experiment (caller time, called samples and calling speed)

Host	Time (s)	# Samples called	# Sample per second
Bare Host	1344	18,614,227,490	1.38475e+07
LPMX	1343	18,614,227,490	1.38643e+07

The host is equipped with an Intel(R) Xeon(R) CPU X5650 @2.67 GHz with 24 cores, GeForce GTX 1080 GPU, 192 GB RAM memory, and its driver info is NVIDIA-SMI: 440.64.00 / Driver ver 440.64.00 / CUDA ver 10.2. We used the Chip137 IVT NA12878 RNA dataset (https://s3.amazonaws.com/nanopore-human-wgs/rna/links/NA12878-IVT-RNA_All.files.txt), which contains 313 fast5 split files with a total size of approximately 35 GB

### Benchmark on importing docker images

Docker can produce a tarred file containing all layers and metadata, which can be imported by other container systems to reproduce environments easily without recreating everything from scratch. Singularity and LPMX can import Docker saved tarballs to recreate the containers by reading the metadata, importing layers, and establishing correct runtimes. Singularity requires additional steps to squash extracted layers to create a read-only Singularity image, while LPMX can utilize incorporated UUFS mentioned in the section to directly mount extracted layers from saved tarballs and is thus more efficient than Singularity, especially when importing many Docker saved tarballs. We measured the import performances of Singularity and LPMX using the same environments mentioned in the section. The versions of all software are the same as those in the previous sections. We randomly selected five Docker images from the Biocontainers repository on the Docker Hub, imported these images using Singularity and LPMX, and measured the time costs by using the Linux built-in *time* command. We ran the experiment five times and calculated the average times. Compared with LPMX, Singularity was 2.5 times slower.

## Discussion

### Limitations

#### Incompatibility with Singularity/Docker

A certain types of Singularity/Docker images do not run in LPMX containers for four reasons: (1) Singularity/Docker can run binaries statically linked with a standard C library; however, LPMX does not work for such binaries because statically linked functions cannot be trapped by the LD_PRELOAD hack that LPMX relies on, and hence, LPMX cannot fake the file system. In practice, binaries statically linked with a standard C library are rarely distributed except for proprietary programs without access to the source code. When the source code is available, recompiling these binaries with a shared standard C library is a recommended workaround. If the source code is unavailable, users can install such statically linked executables on the host and call these executables from inside container by exposing them to LPMX, if needed. (2) A standard C library other than glibc is not tested, although such libraries may work well with LPMX. (3) LPMX does not work with a root account; therefore, this would not be a practical problem. (4) Binaries compiled with Link-Time Optimization (LTO) may not work because of technical issues with the dynamic loader. We are investigating the issues for finding a workaround.

#### Limitations of container systems

Another possible situation that may frustrate users when a container image does not run on a container system is when a standard C library in the container image is significantly newer than the version of glibc on the host. However, this situation happens mainly because newer glibc relies on newer system calls in newer Linux kernels, so Singularity/Docker also have this issue (so LPMX is “compatible” in this case).

#### Limitations due to the nature of pure rootless container systems

(1) The process ID (PID) namespace is not isolated in LPMX as the namepsace is in Docker/Singularity. For example, the ps command shows all processes on the host, whereas the ps in Docker/Singularity shows only processes inside the container. (2) Setuid/setgid executables do not work inside LPMX containers because LD_PRELOAD is disabled by Linux for such executables. (3) Programs cannot open files or devices only accessible by privileged users. (4) Programs cannot listen on privileged ports (below 1024). (5) Programs cannot mount file systems that require privilege on the host. (6) Programs cannot change system settings, including userid, groupid, and system time; network settings, such as firewall rules and interfaces.

#### Practical limitations in genome analysis

These limitations usually would not be a major problem when users execute a genome analysis pipeline with only open-sourced tools that input files and output files without interacting with network sockets; this type of execution is the common case for genome analysis pipelines.

### Security

In contrast with a Docker daemon that has potential security risks, LPMX is a pure rootless, nonsetuid program. Therefore, using LPMX will not increase any security risks by design. Unlike container system implementations that rely on the unprivileged user namespaces technique, LPMX will not increase risks of the host system being compromised via zero-day bugs, for example, this bug [18] in the Linux kernel.

### The necessities of a pure rootless container system on HPC systems

*Rootless* in container technology refers to the ability of unprivileged users to create and manage containers. *Pure rootless*, as defined in the manuscript, refers to the property that privileges are not required even when installing the container system itself and when launching new containers. This property is desired because users can use the same container system at multiple sites regardless of Linux kernels and distributions. Additionally, the attack surface of rapidly evolving software on the host is minimized, and no additional potential risks will be introduced; this situation is suitable for multiple-tenant systems that may have sensitive information such as patients’ genomes.

When a researcher asks administrators to install Docker, the administrators are concerned about security. Docker, which uses a daemon with root privileges, had several critical security bugs [19] in the past. The recent Docker version implements a nonroot mode [20] to reduce such concerns but still suffers security issues from using the unprivileged user namespace. In addition, to enable the nonroot mode, necessary packages, e.g., newuidmap, and fuse-overlayfs, need to be installed in advance; package installations also require root privileges and introduce new security risks. Our system does not require root during both the installation and runtime of containers. The pure rootless design also eliminates security concerns, as the design does not enhance any privileges in userspace. These properties ensure that one can use the same container engine, namely, LPMX, at all sites.

### Future work

Container systems are often used with workflow management systems, and therefore, integrating LPMX into major workflow management systems as an alternative to Singularity/Docker is the next step to take. However, integration in this manner allows users to benefit from the composability of LPMX. Therefore, we need to provide an easy way for the authors of workflow management systems to utilize the composability of LPMX. One possible way to approach this issue is to create a hub (let us call it LPMXHub hereafter). Any workflow management system that we know of allows users to specify a pair of a container system (e.g., Singularity) and a string to specify a container image (e.g., a hash value). Developers write a simple YAML file that describes a composed environment and publish it in LPMXHub, thereby allowing workflow developers to specify a pair of LPMX and the published environment in the workflow description. In this way, workflow management systems would benefit from the composability of LPMX with minimal efforts.

## Conclusions

We developed LPMX, an open-source pure rootless composable container system that provides composability to enable users to easily integrate tools from different containers or even from the host. LPMX saves time by letting researchers compose existing containers and containerize tools that are difficult to package, thereby accelerating science.

## Data Availability

All genomic data were downloaded from public databases, and the origins of the data are indicated in the main text where the data were used.

## Abbreviations

HPC: High-performance computing
CLI: Command line interface
UUFS: Userspace Union File System
GPGPU: General-purpose graphics processing unit
API: Application programming interface
UGE: Univa Grid Engine
HGC: Human Genome Center
GATK: Genome Analysis ToolKit
GIAB: Genome in a Bottle
JRE: Java Runtime Environment

## Footnotes

1 http://nanopore.s3.climb.ac.uk/MAP006-PCR-1_2D_pass.fasta.

2 https://supcom.hgc.jp/shirokane.html.

3 https://app.vagrantup.com/jasonyangshadow/boxes/composability.

4 https://app.vagrantup.com/jasonyangshadow/boxes/benchmark_ubuntu1804.

5 https://github.com/JasonYangShadow/experiment_attachments.

## Declarations

### Availability and requirements

Project name: LPMX; Project home page: https://github.com/JasonYangShadow/lpmx; Operating system(s): GNU/Linux (x86_64); Programming language: Go, C; Other requirements: glibc; License: Apache License 2.0 & GNU LESSER GENERAL PUBLIC LICENSE (ver 2.1); Any restrictions to use by nonacademics: None.

### Software and code

The LPMX source code is open source at https://github.com/JasonYangShadow/lpmx with Apache-2.0 License at https://github.com/JasonYangShadow/lpmx/blob/master/LICENSE.
